# Micro-electron diffraction structure of the aggregation-driving N terminus of *Drosophila* neuronal protein Orb2A reveals amyloid-like β-sheets

**DOI:** 10.1016/j.jbc.2022.102396

**Published:** 2022-08-18

**Authors:** Jeannette T. Bowler, Michael R. Sawaya, David R. Boyer, Duilio Cascio, Manya Bali, David S. Eisenberg

**Affiliations:** Department of Biological Chemistry, UCLA-DOE Institute, Howard Hughes Medical Institute, and Molecular Biology Institute, UCLA, Los Angeles, California, USA

**Keywords:** amyloid, cytoplasmic polyadenylation element binding (CPEB) protein, electron microscopy, functional amyloid, intrinsically disordered protein, micro-electron diffraction (micro-ED), orb2, protein aggregation, protein structure, CPEB, cytoplasmic polyadenylation element binding, LTM, long-term memory, micro-ED, micro-electron diffraction, PLD, prion-like domain, SSNMR, solid-state nuclear magnetic resonance, TEM, transmission electron microscopy, ThT, thioflavin-T

## Abstract

Amyloid protein aggregation is commonly associated with progressive neurodegenerative diseases, however not all amyloid fibrils are pathogenic. The neuronal cytoplasmic polyadenylation element binding protein is a regulator of synaptic mRNA translation and has been shown to form functional amyloid aggregates that stabilize long-term memory. In adult *Drosophila* neurons, the cytoplasmic polyadenylation element binding homolog Orb2 is expressed as 2 isoforms, of which the Orb2B isoform is far more abundant, but the rarer Orb2A isoform is required to initiate Orb2 aggregation. The N terminus is a distinctive feature of the Orb2A isoform and is critical for its aggregation. Intriguingly, replacement of phenylalanine in the fifth position of Orb2A with tyrosine (F5Y) in *Drosophila* impairs stabilization of long-term memory. The structure of endogenous Orb2B fibers was recently determined by cryo-EM, but the structure adopted by fibrillar Orb2A is less certain. Here we use micro-electron diffraction to determine the structure of the first 9 N-terminal residues of Orb2A, at a resolution of 1.05 Å. We find that this segment (which we term M9I) forms an amyloid-like array of parallel in-register β-sheets, which interact through side chain interdigitation of aromatic and hydrophobic residues. Our structure provides an explanation for the decreased aggregation observed for the F5Y mutant and offers a hypothesis for how the addition of a single atom (the tyrosyl oxygen) affects long-term memory. We also propose a structural model of Orb2A that integrates our structure of the M9I segment with the published Orb2B cryo-EM structure.

Amyloid protein aggregation is characterized by the formation of stable, self-propagating, β-sheet rich protein fibrils (1). Although amyloid formation is traditionally associated with neurodegenerative diseases such as Alzheimer’s and Parkinson’s, a growing number of functional proteins have been identified whose function in the amyloid state provides a biological benefit to their host (2, 3). These include components of bacterial biofilms (4, 5, 6) and fungal hydrophobins (7, 8) as well as scaffolding and signaling complexes (9, 10, 11) and several RNA-binding proteins (12, 13, 14, 15). One such functional amyloid RNA-binding protein is the cytoplasmic polyadenylation element binding (CPEB) protein, first shown to have functional amyloid properties in *Aplysia* (*Ap*CPEB) (16). *Ap*CPEB (17), as well as its mammalian homolog CPEB3 (18, 19) and *Drosophila* homolog Orb2 (20, 21), is localized at neuronal synapses and contains the canonical RNA-recognition motifs found in all CPEB proteins, as well as a functional prion-like domain (PLD). As a monomer, CPEB is a repressor of mRNA translation, while synaptic activity promotes formation of stable amyloid-like aggregates of CPEB, resulting in activation of mRNA translation (22, 23). The mRNAs targeted by CPEB are transcripts of several genes that facilitate long-term memory (LTM) persistence (20, 23, 24, 25), and the ability of CPEB to stably maintain an amyloid-like state is proposed as a biochemical mechanism for long-term, synapse-specific changes in protein expression (18, 22, 26).

The *Drosophila* homolog Orb2 has been well characterized, revealing an intriguing aggregation mechanism (25, 26, 27, 28, 29, 30, 31, 32). Two protein isoforms are expressed from the *orb2* gene: Orb2A, which is highly aggregation-prone and kept at extremely low concentration in the resting-state synapse, and Orb2B, which is more soluble and makes up the majority of expressed Orb2 protein. Following synaptic stimulation, Orb2A forms stable aggregates that nucleate Orb2B amyloid formation, thereby switching Orb2 from a translation inhibitor to activator (27, 28, 29), possibly through recruitment of Orb2 monomer- or amyloid-specific binding partners that facilitate RNA degradation or translation, respectively (29, 30). Both Orb2 isoform sequences are nearly identical, consisting of a Q/H-rich region and C-terminal RNA-recognition motifs. The isoforms differ only at the N terminus, whereas Orb2B has a 162-residue serine/glycine-rich N terminus that is predicted to be intrinsically disordered and as of yet has unknown function, and the Orb2A N terminus is only 9-residues in length but is nevertheless critical for its self-assembly (27) and function in initiating Orb2B aggregation (28).

The structure of endogenous Orb2 fibers was recently determined by cryo-EM(32), showing that the ordered fiber core is formed by the Q/H-rich region and is made up of 3 interwound protofilaments, each consisting of paired in-register β-sheets connected by a β-hairpin. Although the Orb2A isoform also contains this Q/H-rich region, several lines of evidence point toward a critical role for the isoform-unique N terminus in Orb2A fiber formation. Deletion or mutation of the Orb2A N-terminal residues was found to reduce formation of insoluble Orb2 aggregates both *in vitro* (31) *and in vivo*, and a single point mutation of the fifth position phenylalanine to tyrosine (F5Y) impaired LTM formation in *Drosophila* (27). Additionally, solid-state NMR (ssNMR) experiments indicate that the N-terminal residues of Orb2A adopt a highly ordered, in-register parallel β-sheet, whereas the Q/H-rich region has more intermediate dynamics and was not required for fiber formation (33).

Here we characterized and determined the structure of the critical nine-residue Orb2A N terminus (which we term M9I) by micro-electron diffraction (micro-ED) and find that this segment forms self-complementary β-sheets driven, at least in part, by hydrophobic aromatic residues F5 and F8.

## Results

We initially characterized full-length Orb2A (Orb2A-FL), and the first 80 residues (Orb2A-PLD) containing both the M9I segment and the Q/H-rich region (Fig. S1, *A* and *E*), following recombinant bacterial protein expression and purification under strong denaturing conditions. Orb2A-FL immediately formed thioflavin-T (ThT)-positive species upon dialysis or dilution into a physiological salt buffer containing mild denaturant (1M urea) (Fig. S1*B*). Transmission electron microscopy (TEM) imaging showed Orb2A-FL forms a heterogenous mixture of fibrillar and amorphous aggregates, and X-ray diffraction analysis of aligned fibers revealed weak broad reflections at ∼4.7 and 10 Å (Fig. S1, *C* and *D*), indicating that Orb2A-FL can form amyloid-like structures, although this may be less efficient *in vitro* without native binding partners (32). In the same incubation conditions, the truncated Orb2A-PLD construct formed ThT-positive species over several days, reaching a much higher endpoint ThT fluorescence relative to Orb2A-FL (Fig. S1*F*); TEM imaging showed that Orb2A-PLD formed abundant, typical amyloid-like fibrils, and these exhibited a similar X-ray diffraction pattern to Orb2A-FL (Fig. S1, *G* and *H*). We attempted to prepare Orb2A-PLD or Orb2A-FL fiber samples suitable for cryo-EM, but fibers remained highly bundled and heterogenous in a variety of buffer and fibrillation conditions.

**Figure 1:**
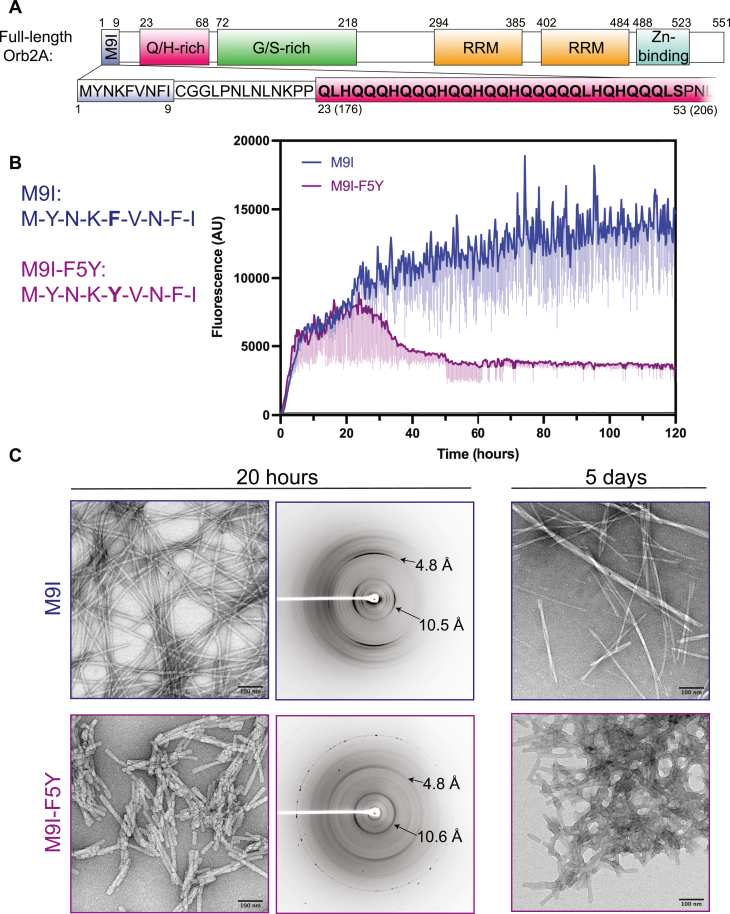
**The hydrophobic nine-residue N-terminal segment of Orb2A (M9I) forms amyloid-like fibers**. *A*, schematic of full-length Orb2A, showing the N-terminal sequence unique to the Orb2A isoform (M9I, *blue*), followed by a 13-residue linker (*white*), and the Q/H-rich domain (*pink*). Both the linker and Q/H-rich domain are common to the Orb2A and Orb2B isoforms. The corresponding residue positions in the Orb2B isoform are in parentheses, and the segment forming the structured core of Orb2B fibers (32)are indicated in *bold*. *B*, left: segment sequences for wildtype M9I (*blue*) and M9I with the F5Y mutation (*purple*). Right: kinetic thioflavin T (ThT) assay comparing aggregation kinetics of wildtype M9I and M9I-F5Y at 1 mg/ml concentration (∼850 μM). The darker line represents the average reading of 3 independent technical repeats, and the lighter vertical bars below represent 1 standard deviation. *C*, left: negative-stain TEM analysis of fibers formed by M9I and M9I-F5Y after 20 h of incubation under identical conditions as (panel B) but without added ThT. Middle: X-ray diffraction of aligned dried fibers at 20 h incubation. Right: negative-stain TEM analysis of M9I and M9I-F5Y fibers after 5 days incubation. RRM, RNA-recognition motif.

We therefore chose to focus on the short but critical M9I segment for our high-resolution structural studies (Fig. 1*A*). When incubated in physiological salt buffer at room temperature, M9I forms amyloid fibrils within a few hours, reaching maximum ThT fluorescence within ∼1 to 2 days (Fig. 1*B*, blue). TEM imaging shows that M9I forms twisting, unbranched fibers approximately 10 nm in width and up to several μm in length. Aligned M9I fibers exhibited the amyloid-characteristic cross-β diffraction pattern, with sharp meridional reflections at 4.8 Å and equatorial reflections at 10.5 Å (Fig. 1*C*, top row).

We also tested the effect that the F5Y mutation has on M9I amyloid formation. When incubated under the same conditions, M9I-F5Y appears to initially bind similar levels of ThT as the wildtype (WT) M9I peptide, but the signal drops after ∼1 day and remains significantly lower than WT peptide by the end of 5 days incubation (Fig. 1*B*, purple). Examination by TEM at 20 h shows that M9I-F5Y forms rod-like fibers which appear slightly wider and significantly shorter than M9I fibers, measuring only ∼50 to 300 nM in length (Fig. 1*C*, left). Fiber morphology remained similar after 5 days of incubation (Fig. 1*C*, right), and while individual M9I fibrils tended to assemble into thicker rope-like filaments, M9I-F5Y fibers remained short and aggregated into large clumps. The drop in ThT fluorescence after ∼1 day for M9I-F5Y may be due to the ThT-binding site becoming occluded as the fibers become clumped or a conversion over time to an alternate aggregate structure with poor ThT-binding. X-ray diffraction of M9I-F5Y fibers after 1 day incubation revealed powder diffraction-like rings (Fig. 1*C* middle), consistent with poor alignment of these short fibers. Additionally, lower resolution reflections differ significantly between M9I and M9I-F5Y fibers (Fig. S2), which may indicate an altered arrangement of pairs of β-sheets within individual protofibrils. Taken together, these results show that the M9I segment is sufficient to rapidly and efficiently form amyloid-like fibers, and incorporation of the F5Y mutation results in an altered morphology that appears to preclude the formation of typical well-ordered, elongated amyloid fibers.

**Figure 2:**
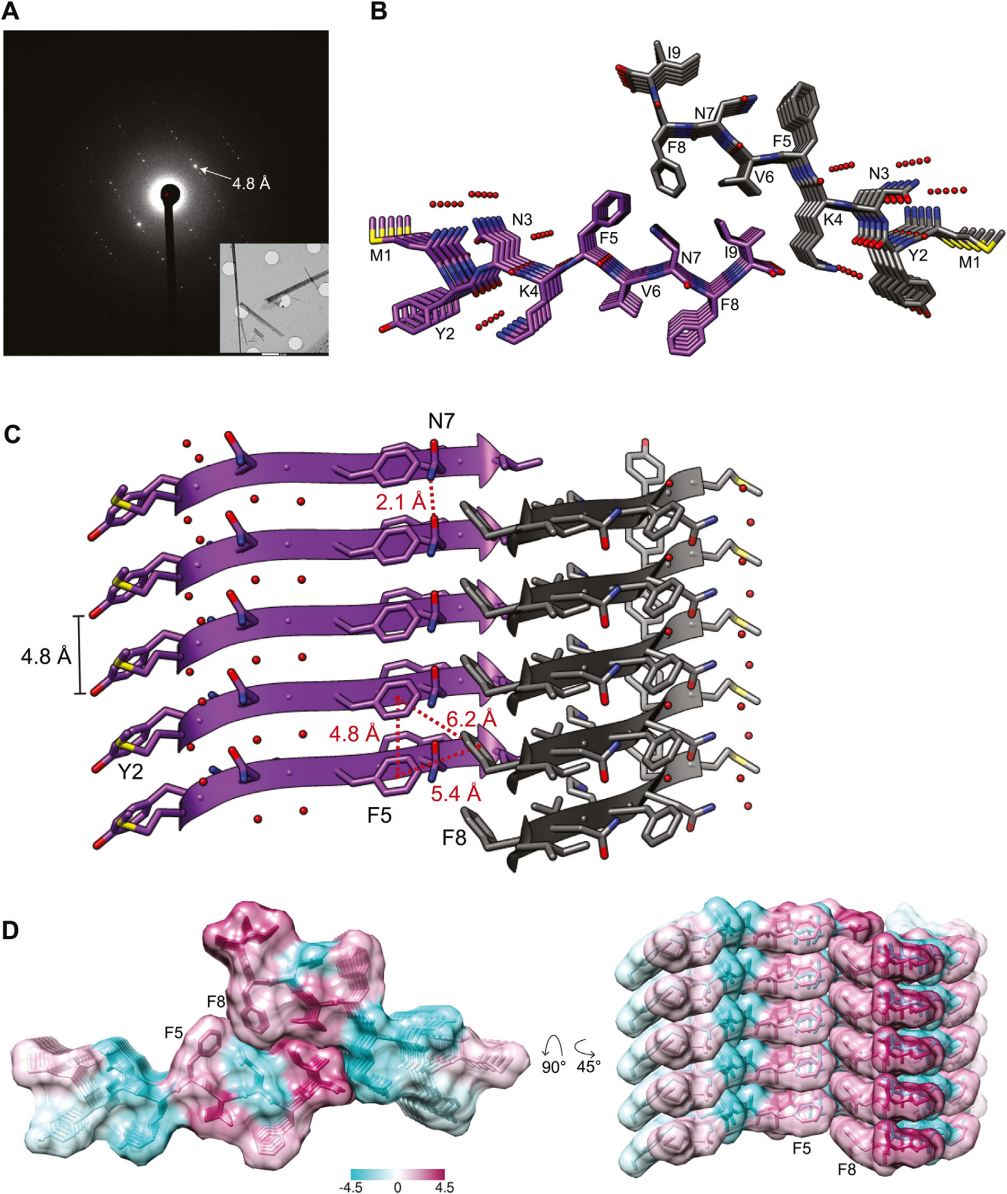
**Structure of M9I segment as determined by micro-electron diffraction**. *A*, representative electron diffraction pattern from micro-ED data collection on M9I microcrystals. Strong reflections at 4.8 Å (*white arrow*) correspond to inter-β-strand spacings. Inset: electron micrograph of M9I microcrystals on Quantifoil grids; scale bar: 2 μm. *B*, structural model of M9I shows formation of in-register parallel β-sheets. Two sheets are viewed down the fibril axis, illustrating the water-excluded interface formed between sheets. Strands are related to each other *via* a 2_1_-screw axis perpendicular to the fiber axis (class 4 steric zipper). *Red spheres* represent ordered water molecules. *C*, view perpendicular to fibril axis, showing side chain stabilizing interactions along the fibril axis. Asparagine 7 (N7) side chain forms a ladder of hydrogen bonds, and aromatic residues (Y2, F5, F8) stack in a parallel-displaced fashion. F5 and F8 also interact closely in the dry interface. Atomic separations are indicated in red; distances between aromatic side chains were calculated using the benzyl ring centroid point (*red stars*). *Red spheres* represent ordered water molecules. *D*, space-filling model of M9I colored according to the Kyte-Doolittle hydrophobicity scale (*magenta*-hydrophilic, *teal*-hydrophobic), showing tight packing of hydrophobic residues in the intersheet interface.

We next determined the atomic structure of M9I in the fibrillar state at 1.05 Å resolution using micro-ED. Crystallization screening of the M9I peptide showed a propensity to form small fibrillar and needle-like crystals, but single crystals could not be grown large enough for traditional X-ray data collection (Fig. S3). However, examination of the hanging drop crystallization solution by TEM revealed microcrystals of an optimal size for micro-ED, which has previously been used to solve the structures of other amyloid spine segments (34, 35, 36). We grew microcrystals in batch by incubating equal volumes of peptide stock solution with crystallization solution, and prepared sample grids using a similar workflow to single-particle cryo-EM. M9I microcrystals diffracted to high resolution (resolution cutoff at 1.05 Å), and the phases were determined using direct methods (Fig. 2*A*, Table 1).

**Table 1:** Statistics of micro-ED data collection and atomic refinement

Data collection	
Excitation voltage (kV)	200
Electron source	Field emission gun
Wavelength (Å)	0.0251
Space group	P2_1_
Unit cell dimensions	
*a, b, c* (Å)	4.83, 23.1, 29.8
α, β, γ (°)	90.0, 92.0, 90.0
Resolution (Å)	18.26–1.05 (1.08–1.05)^a^
R_merge_ (%)	15.6 (71.2)
Measured reflections	36,014 (1101)
Unique reflections	2596 (153)
Completeness (%)	83.3 (69.9)
Multiplicity	13.9 (7.2)
I/σ	8.24 (1.79)
CC_1/2_ (%)	99.4 (57.2)
Refinement	
Reflections in working set	2336
Reflections in test set	260
R_work_ (%)	18.1
R_free_ (%)	20.1
RMSD bond length (Å)	0.01
RMSD angle (°)	1.84
Number of non-H atoms in refinement	87
Average B-factor (Å^2^)	10.7
Ramachandran (%)	
Favored	100
Allowed	0
Outliers	0

a Highest resolution shell shown in parentheses.

Our structural model reveals that M9I forms an array of parallel, in-register β-sheets arranged in a face-to-back orientation, as defined by a class 4 steric zipper. Aromatic side chains (Y2, F5, F8) are stacked in an energetically favorable parallel-displaced orientation (37) along the fiber axis (Fig. 2*B*), and in addition to main chain hydrogen bonds, a hydrogen-bonded ladder forms parallel to the fibril axis between amide side chains of N7 (Fig. 2*C*). The C-terminal residues F5-I9 assemble into a water-excluded steric zipper, in which the F5 benzyl ring stacks in an edge-to-face orientation against F8 of the neighboring sheet. In contrast to the tightly packed interface formed by C-terminal residues, the N-terminal residues M1-K4 are more loosely packed and hydrated, with side chains interacting with a network of ordered water molecules (Fig. S4*B*). We predict that in full-length Orb2A fibers, these N-terminal residues are either solvent exposed or may interact with polar residues further downstream of the M9I segment. The average solvation energy per residue upon assembly of the M9I dry interface was calculated to be -0.56 kcal/mol, on par with solvation energies of formation of pathogenic amyloid steric zippers (14). The dry interface buries relatively little surface area (128.2 Å^2^) compared to steric zippers on average (∼150–200 Å^2^) (1), but the high calculated shape complementary (0.88) reflects the close packing and favorable van der Waals contacts formed by hydrophobic side chains (Fig. S4*C*).

**Figure 3:**
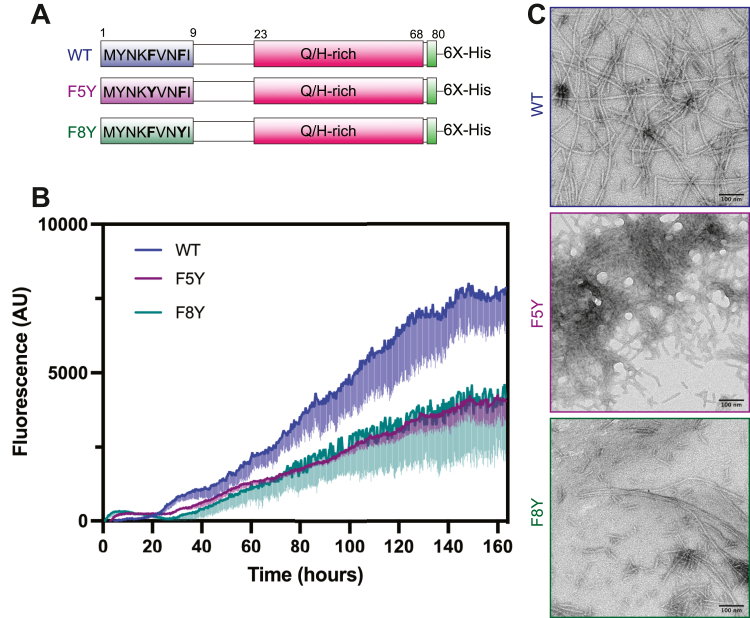
**Phenylalanine replacements reduce fibrillation of Orb2A prion-like domain**. *A*, schematic of Orb2A prion-like domain (PLD) constructs (residues 1–80) with either the fifth (F5Y) or eighth (F8Y) phenylalanine replaced by tyrosine. *B*, ThT assay comparing aggregation of 10 μM wildtype PLD (*blue*) to the F5Y (*purple*) or F8Y (*green*) PLD constructs. Samples were incubated at 25 °C in Hepes-KCl-urea pH 7.4 buffer. The *darker line* represents the average reading of technical triplicates, and the *lighter vertical bars* represent 1 standard deviation. *C*, negative stain TEM imaging of WT, F5Y, and F8Y Orb2A-PLD after 7 days incubation in identical conditions as above, but without ThT added. ThT, thioflavin-T; TEM, transmission electron microscopy.

Our structure offers a hypothesis for the effect of the F5Y amino acid substitution, as addition of a hydroxyl group would cause a steric clash with F8 on the neighboring sheet, potentially destabilizing the intersheet interface. We further explored the contribution of the F5-F8 interaction, as intersheet Phe–Phe interactions have been reported to play a role in driving fiber formation of other amyloidogenic proteins (38). We expressed and purified the OrbA-PLD (residues 1–80) with either F5 or F8 replaced by tyrosine (Y) to sterically disrupt the packing of these residues in the dry interface and compared aggregation kinetics and fiber morphology to the WT Orb2A-PLD (Fig. 3). Whereas WT Orb2A-PLD formed abundant elongated fibrillar species, the F5Y variant formed short fibrils reminiscent of those formed by the M9I-F5Y segment and exhibited a corresponding reduction in ThT fluorescence. The F8Y variant appeared to form a heterogenous mixture of long and short fibers, although reduced ThT fluorescence indicates less efficient fibril formation. Taken together, these results show that the M9I segment of Orb2A adopts a parallel, in-register steric zipper structure with side chains of F5 and F8 stacked within the dry intersheet interface and that replacement of either of these residues impairs formation of amyloid-like fibrils of the Orb2A-PLD.

**Figure 4:**
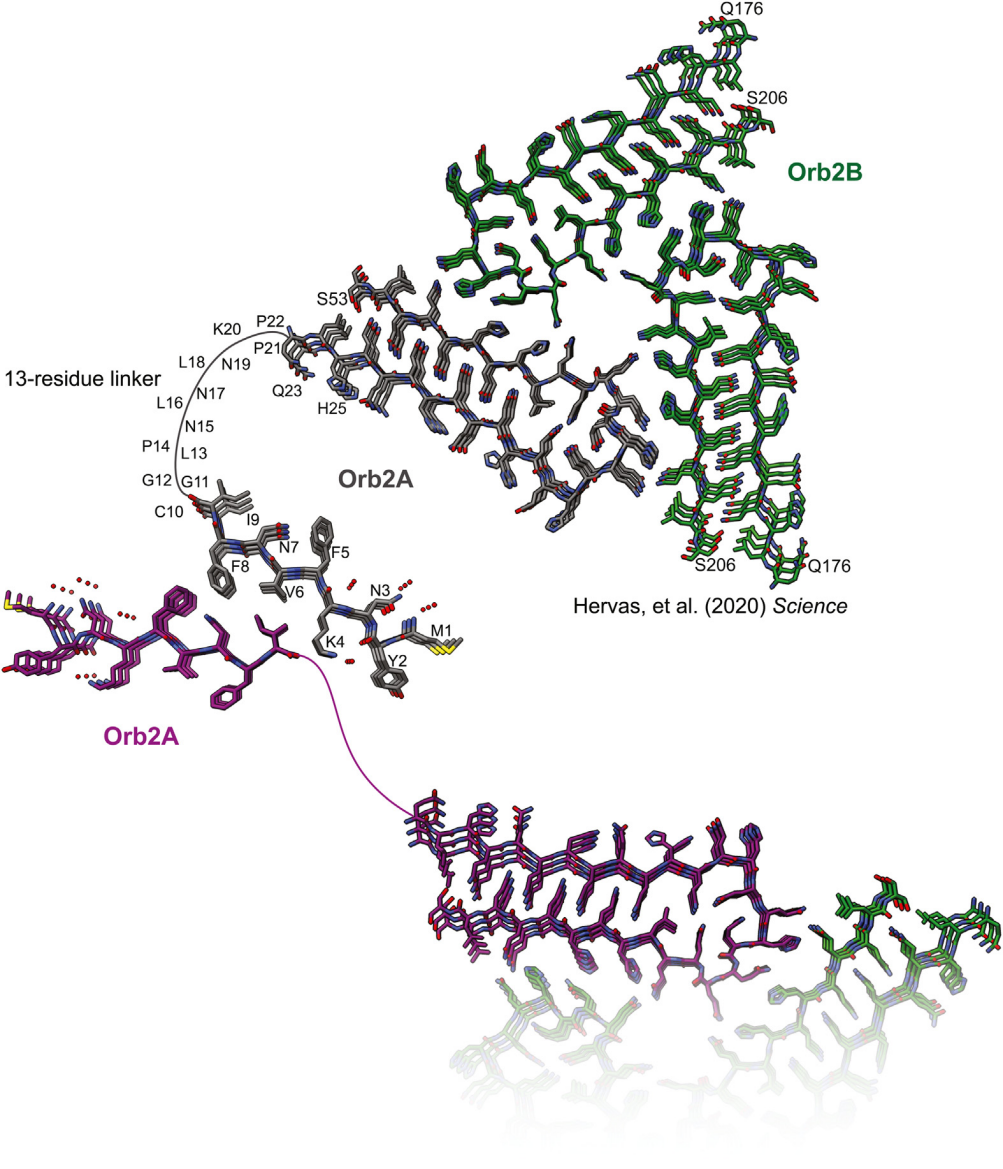
**Proposed structural model of an Orb2A–Orb2B heterocomplex**. Aggregation of Orb2A (gray and purple) may initially be driven by steric zipper formation of the hydrophobic N-terminal M9I segment. The downstream Q/H-rich region, connected by a disordered 13-residue linker, would then be oriented in-register and can adopt an identical protofilament structure as that formed by endogenous Orb2B fibers (32). Formation of such an Orb2A protofibril seed could then nucleate Orb2B (*green*) fibril formation *via* structurally homotypic seeding of their identical Q/H-rich regions.

We also examined whether it is sterically possible for the Orb2A Q/H-rich region, which is connected to M9I *via* a 13-residue linker (Fig. 1), to adopt a protofilament structure of the form of endogenous Orb2B fibers (32). In both structures, individual strands are oriented in parallel in-register β-sheets, and extension of a pair of sheets from the M9I structure shows that the Q/H-rich region of Orb2A can potentially form the hairpin-like amyloid fold of Orb2B(32), connected *via* the flexible 13-residue linker (Fig. 4). The orientation of paired β-sheets in the class 4 M9I steric zipper would result in an asymmetric orientation of the hairpin-like fold of the downstream Q/H-rich region: one strand (colored gray) can potentially form a more compact serpentine-like structure, in which F5 may interact with the exposed outer interface of the β1 strand of the Q/H-rich region of Orb2A. The mated Orb2A strand (colored purple) would be sterically hindered from forming a similar serpentine-like fold and would need to adopt a more extended conformation to accommodate the hairpin-like fold of the Q/H-rich region.

## Discussion

Prior structural studies of Orb2A indicate that the protein can adopt multiple conformations but have left unclear the structure of the isoform-unique M9I segment. *In vitro*, Orb2A is reported to form parallel in-register β-sheets over time (31, 33, 39), with one ssNMR study indicating that the M9I segment, but not the Q/H-rich region, forms the ordered fiber core (33). Orb2A can also undergo liquid–liquid phase separation and subsequent fiber formation, although analysis of immobile residues in that study did not clearly point to the presence of M9I segment (40). In a solution-state NMR study of the Orb2A PLD, the Q/H-rich region adopted varying degrees of α-helical secondary structure, whereas the rest of the protein, including M9I, remained disordered (41), and in the presence of lipids, the N terminus was found to form an α-helix and Orb2A fibrillation was inhibited (42). The M9I segment was not observed in the endogenous Orb2 cryo-EM structure (32), but this may be expected given that the Orb2B isoform is predominantly expressed. Given the critical role of the M9I segment in initiating Orb2 aggregation, determining its fibrillar structure would improve our understanding of the early steps in Orb2 amyloid formation.

To this end, we used micro-ED to determine the atomic resolution structure of the M9I segment from Orb2A and show that a hydrophobic stretch of residues (5-FVNFI-9) forms a self-complementary steric zipper core, consistent with ssNMR data of the Orb2A PLD(33). Phenylalanine residues (F5 and F8) of neighboring sheets stack closely within the dry interface, leading us to hypothesize that the previously characterized F5Y mutation (27, 31) may reduce Orb2A amyloid formation by sterically hindering proper assembly of β-sheets, instead forcing assembly through an alternative interface. Whereas our X-ray diffraction results indicate that strands formed by M9I-F5Y (Fig. 1) are likely still oriented in a cross-β arrangement (although not necessarily a class 4 zipper), ThT assays suggest that fibrillation is less efficient, and EM imaging of fiber assemblies show the mutation induces a distinct morphology relative to WT fibers. Screening crystallization conditions for M9I-F5Y yielded only amorphous aggregates or very small fibrous species (Fig. S3*B*), and we suspect that the F5Y mutation may preclude formation of a well-ordered crystalline lattice large enough to be useful for structure determination.

Examination of the M9I structure reveals stabilizing interactions that are common among amyloid-driving steric zipper segments. Aromatic residues, of which M9I has 3, have been proposed to stabilize amyloid protein assembly (38, 43, 44, 45, 46, 47, 48), and here we find in-register stacking of aromatic side chains produces favorable π–π interactions along the fiber axis (Fig. 2*C*). In addition, neighboring β-sheets interact through a self-complementary interface made up primarily of aromatic and hydrophobic side chains, and polar resides (2-YNK-4) interact with ordered water molecules. Only one polar residue, N7, is part of the dry interface and forms a hydrogen-bonded ladder of stacked amide side chains along the fiber axis. Such polar ladders frequently form between stacked side chains parallel to the amyloid fiber axis (48, 49, 50, 51, 52, 53, 54, 55), including in the endogenous Orb2B structure (32).

Additionally, energetic stability calculations reveal that the M9I steric zipper structure is of relatively similar energetic stability (14) and shape complementarity (1) to pathogenic amyloid zippers, which may in part explain the strong amyloid-forming propensity of this segment. *In vivo*, aberrant Orb2A aggregation may be avoided by regulation of protein expression levels, *e.g.,* by posttranslational modifications that affect protein stability (29) and variations in intron splicing (56). Orb2B fibers are also unstable under acidic conditions allowing for lysosomal degradation (32), and Orb2 fibrillation may also be modulated by lipid membranes (42) and divalent metal binding (39).

On the basis of our structure of M9I, we propose a structural model for full-length Orb2A (Fig. 4) that integrates the endogenous Orb2 fiber core structure (32) and is consistent with functional studies demonstrating that the more aggregation-prone Orb2A is required for initiating conversion of soluble monomeric Orb2B into stable aggregates (25, 27, 28, 29, 30). We predict that Orb2A aggregation is initially driven by the hydrophobic, steric zipper–forming propensity of the isoform-unique M9I segment. Formation of the M9I steric zipper structure would orient multiple strands of the adjacent Q/H-rich region in close proximity, effectively increasing the local concentration and promoting conversion to a more stable β-sheet conformation. Orb2A may then seed Orb2B by templating hydrogen-bonded stacking of their common Q/H-rich regions. Such structurally homotypic seeding is the most common mechanism by which amyloid fibers grow (1, 57), and the Q/H-rich region is known to be critical for Orb2B recruitment into synaptic complexes by Orb2A (27, 28). Although such a complex is structurally feasible, it is highly speculative at this point, and further studies will be required to determine if it describes the Orb2 aggregation mechanism.

In conclusion, our results add to the growing literature on the functional amyloid activity of Orb2 and suggest a plausible structural mechanism for initiation of Orb2 aggregation. Ultimately, this work may aid in our understanding of LTM maintenance and yield insights into the molecular mechanisms of amyloid formation and disease.

## Experimental procedures

### Plasmids and cloning

Orb2A-FL plasmid was a gift from Dr Ansgar Siemer (USC), expressing full-length Orb2A in a pET-28 vector with a C-terminal His-tag. The Orb2A-PLD construct was generated using overlap-extension PCR (58) and the F5Y and F8Y mutants were generated using the Quikchange II site-directed mutagenesis kit (Agilent Technologies Inc). All plasmid sequences were confirmed by Sanger sequencing (Laragen Inc).

### Protein expression and purification

Recombinant protein was expressed and purified largely following previously established protocols (33). Plasmids were transformed into Bl21-Gold(DE3) cells (Agilent) and cultures grown at 37 °C in LB-kanamycin media until *A*_600_ reached ∼0.6, then expression is induced with 1 mM IPTG and cultures incubated either at 30 °C for 4 to 5 h (Orb2A-FL) or 20 °C for 15 to 18 h (Orb2A-PLD constructs). Cell pellets were harvested at 4,000*g* for 15 min at 4 °C and stored at -80 °C until use. Orb2A-FL and Orb2A-PLD constructs were solubilized by different methods as follows: FL pellets were resuspended in FL-lysis buffer (50 mM Tris pH 8.0, 100 mM NaCl, 0.5% (v/v) Triton X-100, 0.05% (v/v) 2-mercaptoethanol) supplemented with Halt protease inhibitor (ThermoFisher) and sonicated on ice for ∼10 min, followed by centrifugation at 10,000 rpm (Sorvall SS-34 rotor) for 15 min at 4 °C. The soluble fraction was discarded, and the insoluble fraction re-suspended in FL-extraction buffer (100 mM sodium phosphate pH 8.0, 250 mM NaCl, 6 M guanidine hydrochloride, 10% (v/v) glycerol, 1 mM DTT), sonicated again, then incubated overnight on an orbital shaker at room temperature; the next day, the soluble fraction was separated by centrifugation at 15,000 rpm for 30 min at 4 °C. PLD construct pellets were resuspended in PLD-lysis buffer (10 mM Tris pH 8.0, 8 M urea, 100 mM sodium phosphate, 0.05% (v/v) 2-mercaptoethanol, Halt protease inhibitor) and sonicated on ice for ∼15 min, followed by centrifugation at 15,000 rpm for 30 min at 4 °C. The remaining purification steps are the same for all constructs: the soluble cell lysate was filtered thru a 0.45-μm high-particulate syringe-driven filter (HPF Millex-HV, Millipore). A 5-mL HisTrap-HP column (GE Healthcare) was equilibrated in buffer A (10 mM Tris pH 8.0, 8M urea, 100 mM sodium phosphate, 1 mM DTT), and filtered lysate was loaded at 1 ml/min. The column was washed with buffer A + 0.5% (v/v) Triton X-100, then buffer A + 500 mM NaCl, then buffer A adjusted to pH 6.7, and finally buffer A alone again. Orb2A protein was then eluted with a step gradient of elution buffer (buffer A + 500 mM imidazole pH 8). Most protein eluted at ∼100 mM imidazole. Purified protein was loaded into 6000 to 8000 molecular weight cutoff dialysis tubing (Fisher Scientific) and dialyzed at 4 °C against PBS pH 7.4, 4 M urea, 10% (v/v) glycerol, 1 mM DTT, then filtered thru a 0.2-μm spin filter (Millipore). Protein concentration was measured by A280 absorbance using a NanoDrop One (ThermoScientific) and the calculated extinction coefficient and snap frozen in liquid nitrogen and stored at -80 °C.

### Peptide preparation

Peptides were purchased from Genscript at >98% purity and stored in lyophilized form at -20 °C. For all experimental procedures, peptides were first weighed out and solubilized in 100% DMSO (Fisher Bioreagents cat. #BP231100), then further diluted with milliQ water or buffer to the desired final concentration.

### ThT assays

ThT assays were performed in black polystyrene 96-well plates (ThermoFisher cat. #265301) sealed with UV optical tape, with a total volume per well of 200 uL. ThT fluorescence was measured with an excitation/emission wavelength of 440/480 nm using a FLUOstar Omega plate reader (BMG LABTECH), and readings were taken every 15 min. Experiments were performed at 25 °C, without agitation except for a 5 s, 300 rpm shake directly before each reading. ThT curves are averaged from 3 independent replicates with error bars showing standard deviation. For M9I-WT and M9I-F5Y, peptides were dissolved in 100% DMSO (Fisher Bioreagents cat. #BP231100), then diluted in PBS pH 7.4, and filtered with a 0.2 μM spin filter (Millipore). Peptide solution was then mixed with ThT stock solution (100 μM ThT in 1× PBS pH 7.4) in 96-well plates, yielding a final concentration of 1 mg/ml peptide (∼850 μM) in PBS pH 7.4 (1% (v/v) DMSO) and 20 μM ThT. For Orb2A-FL and Orb2A-PLD (Fig. S1), protein stocks were diluted to 10 μM in PBS pH 7.4, 1 M urea, 1 mM EDTA, 5% (v/v) glycerol, 1 mM DTT, and 10 μM ThT. For Orb2A-PLD mutant ThT assays (Fig. 3), protein stocks were diluted to 10 μM in 10 mM Hepes pH 7.6, 100 mM KCl, 1 M urea, 0.1 mM CaCl2, 1 mM MgCl2, 5% (v/v) glycerol, 1 mM DTT, and 10 μM ThT.

### Transmission electron microscopy

Protein and peptide samples were prepared for TEM in 96-well plates as described above for ThT assays, except that ThT dye was not added (instead an equal volume of PBS pH 7.4 was added). Samples were spotted onto freshly glow-discharged carbon-coated formvar grids (Ted Pella Inc) and allowed to adsorb for 3 to 4 min before wicking off excess liquid with Whatman filter paper. The grids were then washed twice with milliQ water, followed by staining with 2% (w/v) uranyl acetate for 2 min, wicked off, and the grids allowed to fully dry before imaging. Grids were imaged using either a T12 or T20 electron microscope (FEI).

### Fiber diffraction

Orb2A-FL and Orb2A-PLD stocks were diluted to 10 μM in PBS pH 7.4, 1 M urea, 1 mM EDTA, 5% (v/v) glycerol, and 1 mM DTT and incubated at room temperature without agitation; Orb2A-FL was incubated for 1 day and Orb2A-PLD for 7 days. Fibrils were spun down in a tabletop microcentrifuge at 21,000*g* for 20 min. The supernatant was removed, and fibers were gently resuspended in milliQ water followed by centrifugation to remove salt from the sample. Pelleted fibers were then resuspended in milliQ water to yield a 50× concentrated sample (relative to starting volume) and suspended between 2 glass capillary ends and let to dry overnight. Fiber diffraction images were taken with a RIGAKU R-AXIS HTC imaging plate detector using CuK_α_ radiation from a Rigaku FRE+ rotating anode generator with VARIMAX HR confocal optics (Rigaku). Radial profiles were calculated with a program written in-house that calculates the average intensity as a function of distance from the beam center.

### M9I crystallization and structure determination by micro-ED

Micro-ED data collection and processing was performed essentially as described in previously established protocols (59, 60). Lyophilized M9I peptide was dissolved in 100% DMSO (ThermoFisher), followed by dilution with milliQ water to yield a 6 mg/ml peptide solution in 1% (v/v) DMSO, then filtered using a 0.2-μm spin filter (Millipore). The filtered solution was mixed in a 1:1 ratio with a 0.1 M sodium phosphate pH 4.6, 1.5 M NaCl solution and allowed to incubate quiescently on the benchtop at room temperature, and microcrystals grew within 2 to 3 days; 4 uL of microcrystal solution was dispensed onto freshly glow-discharged Quantifoil Cu R1/4300-mesh carbon grids and allowed to adsorb for 4 min before wicking off excess liquid, and grids were washed twice with milliQ water to remove excess salt. Grids were plunge frozen into liquid ethane using a Vitrobot Mark IV (FEI) and either stored in liquid nitrogen until use or placed into a cryogenic autoloader for imaging. Images and diffraction data were collected using a Thermo Fisher Talos Arctica cryo-electron microscope operating at 200 kV and fitted with a Ceta-D CMOS 4k x 4k camera. Images were recorded as a movie in rolling shutter mode with 2 x 2 pixel binning (61), and an exposure time of 2 s per frame, while the stage was continuously rotated within the electron beam at a fixed rate of 0.3° per s, corresponding to a fixed angular wedge of 0.6° per frame. Datasets spanned a wedge of reciprocal space ranging from 40 to 90°. We used a selected area aperture with an illuminating spot size of ∼1 μM, thereby equating to a total electron dose rate of <0.01 e^-^/Å^2^ per s deposited onto our crystals. Diffraction movies were converted from SER files to SMV format using publicly available software (https://cryoem.ucla.edu/pages/MicroED) (59). Diffraction images were indexed and integrated with XDS, and datasets scaled and merged with XSCALE (62) from 11 different crystals. Phases were determined by direct methods with SHELXD (63). Subsequent rounds of model building and refinement were performed using COOT (64) and REFMAC (65), respectively. Electron scattering factors were used for refinement. Data processing and refinement statistics are reported in Table 1.

### Structure stability calculations

Surface area buried and shape complementarity (Sc) were calculated using AREAIMOL (66, 67) and Sc (68, 69, 70), respectively. Solvation energy was calculated based on previously published work (14, 43, 71, 72).

All graphs were made with GraphPad Prism, version 9. Diffraction images were analyzed using the Adxv software package (Scripps). All M9I structure images were made using either PyMol or UCSF Chimera; hydrophobicity gradients were generated in Chimera based on the Kyte-Doolittle scale (73). Atomic distances were measured using Chimera; for aromatic residues, the centroid of the benzene ring was used as the point of reference.

## Data availability

The atomic coordinates and structure factors have been deposited in the Protein Data Bank (http://wwpdb.org/) Accession code: 7SXN.

## Supporting information

This article contains supporting information.

## Conflict of interest

D.S.E. is a SAB member and equity holder in ADRx, Inc.
