# Supplementary material for: Micro-electron diffraction structure of the aggregation-driving N terminus of Drosophila neuronal protein Orb2A reveals amyloid-like β-sheets
Source: J Biol Chem. 2022 Aug 18;298(10):102396. doi: 10.1016/j.jbc.2022.102396 (PMC9556795; doi:10.1016/j.jbc.2022.102396)
Supplement: Supporting Information [file mmc1.pdf]

Supporting Information

Micro-electron diffraction structure of the aggregation-driving N-terminus of *Drosophila* neuronal protein Orb2A reveals amyloid-like β-sheets

Jeannette T. Bowler[a,*], Michael R. Sawaya[a], David R. Boyer[a], Duilio Cascio[a], Manya Bali[a], David S. Eisenberg[a,*]

[a]*Department of Biological Chemistry, UCLA-DOE Institute, Howard Hughes Medical Institute, and Molecular Biology Institute, UCLA, Los Angeles, California, USA*

*for correspondence: jbowler@ucla.edu (JTB), david@mbi.ucla.edu (DSE)

Figures S1-S4

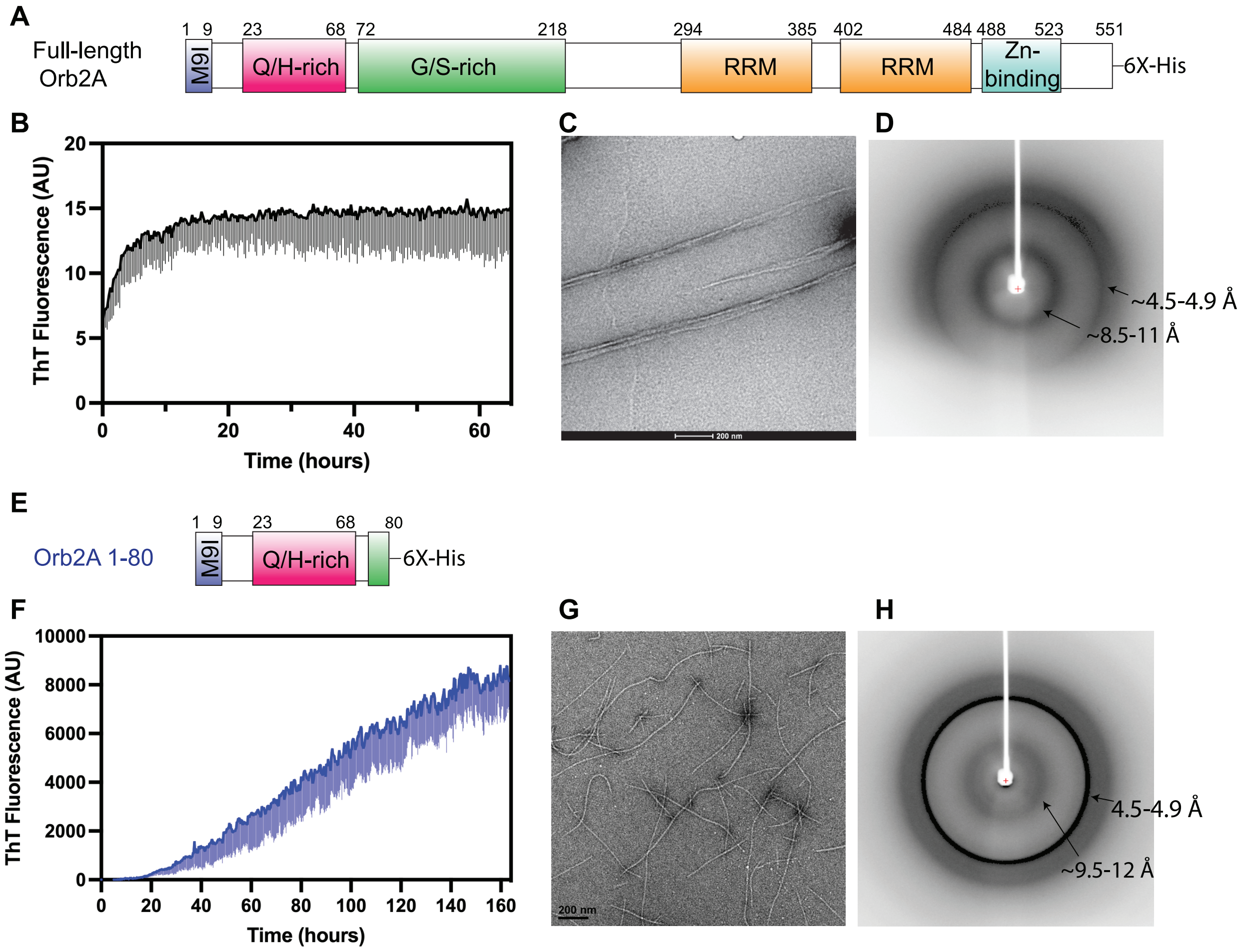

Fig. S1: Amyloid fibril formation of Orb2A-FL and Orb2A-PLD

(**A**) Schematic of recombinant Orb2A-FL construct (**B**) ThT assay of 10 μM Orb2A-FL at 25 °C. The darker line represents the average reading of technical triplicates, and the lighter vertical bars represent 1 standard deviation. (**C**) Negative stain TEM imaging of FL-Orb2A after 1 day incubation as in (panel B), but without ThT added. (**D**) Fiber diffraction on dried, aligned Orb2A-FL fibers prepared as in (panel C). (**E**) Schematic of recombinant Orb2A-PLD construct (**F**) ThT assay of 10 μM Orb2A-PLD at 25 °C. The darker line represents the average reading of technical triplicates, and the lighter bars represent 1 standard deviation. (**G**) Negative stain TEM of imaging Orb2A-PLD after 7 days incubation as in (panel F) but without ThT added. (**H**) Fiber diffraction on dried, aligned Orb2A-PLD fibers prepared as in (panel G).

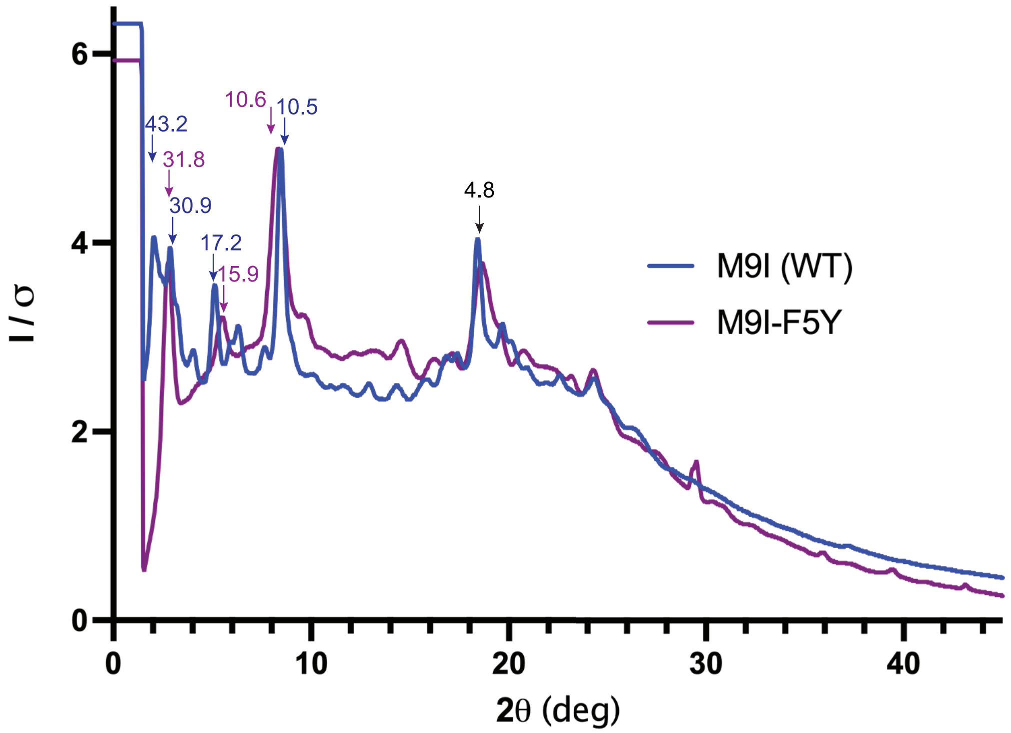

Fig. S2: Radial profile comparison of M9I and M9I-F5Y fiber diffraction

For M9I and M9I-F5Y fiber diffraction patterns (Fig. 1C) the average signal-to-noise ratio (I/σ) was plotted as a function of distance from beam center (2θ). Values indicated above reflection peaks indicate corresponding separations in real space (Å). Both M9I and M9I-F5Y exhibit reflection peaks at 4.8 Å and 10.5/10.6 Å respectively, indicative of an underlying parallel β-sheet structure, while lower resolution reflections (~11-45 Å) differ significantly, suggestive of altered packing between pairs of β-sheets.

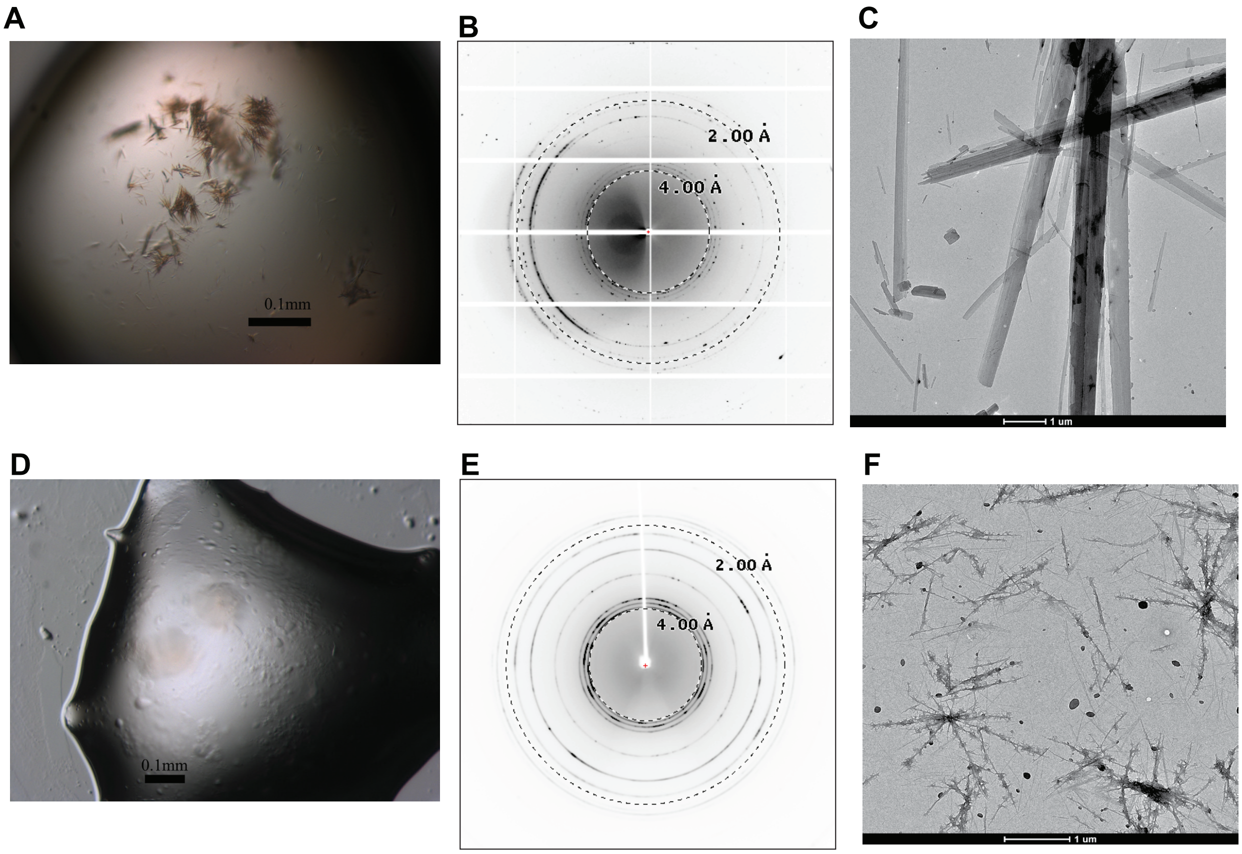

Fig. S3: Crystallization of M9I and M9I-F5Y

(**A**) M9I forms clusters of needle-like microcrystals that are visible by light microscopy in hanging drop vapor diffusion experiments, but could not be grown larger by optimization/seeding. Crystallization condition: 6 mg/mL M9I peptide dissolved in 1% DMSO (in milliQ water) mixed 1:1 with reservoir solution (100 mM sodium phosphate pH 4.6, 2.0 M NaCl) and incubated at 10 °C for 2 weeks. Crystals formed after 2-4 days incubation. (**B**) Microcrystals were looped and cryogenically frozen, and analyzed using the microfocus beamline (24-ID-E) at the Advanced Photon Source (APS). Individual crystals were difficult to find, and x-ray analysis showed no visible diffraction in the range typical for peptides (~5-30 Å) (spots are due to ice contamination). (**C**) TEM imaging of M9I hanging drop experiments show abundant formation of microcrystals up to ~1 μm in width and several μm in length; larger microcrystals tended to contain multiple overlapping lattices when analyzed by micro-ED, and the best diffraction was obtained from smaller single microcrystals with a rectangular shape. (**D**) M9I-F5Y was set up in hanging drop crystallization trays under the same conditions as in (panel A) but formed only amorphous aggregates, even after extended (months) incubation. (**E**) M9I-F5Y aggregates were looped and analyzed using an in-house x-ray source; only ice

diffraction was observed. (**F**) TEM imaging of M9I-F5Y aggregates from hanging drop experiments shows that the segment forms short, narrow fibrillar species, in contrast to the relatively large microcrystals formed by wild-type M9I in the same condition (panel C). Optimization around this condition, or other hits identified for M9I-F5Y in high-throughput screens, did not induce formation of ordered crystals.

A

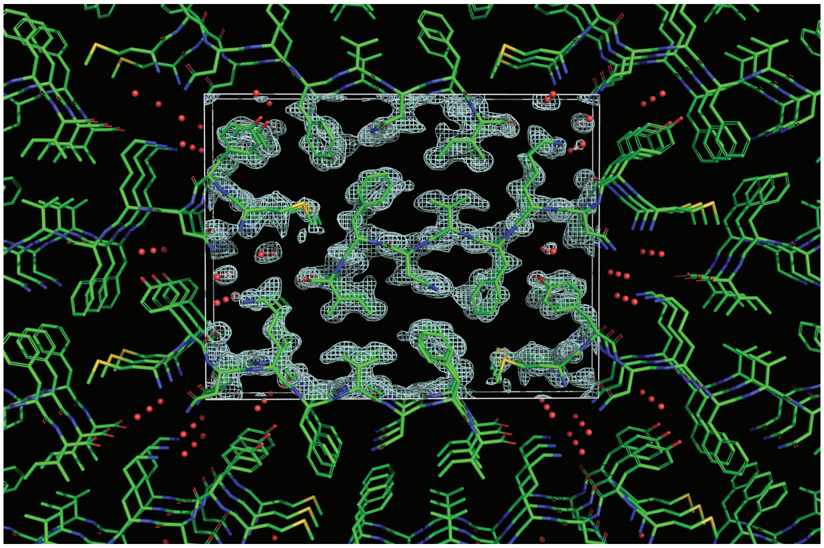

B

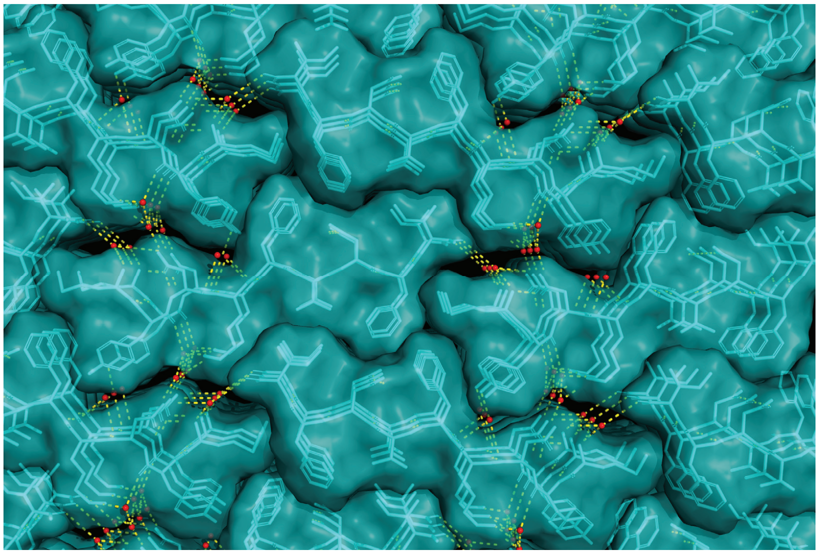

C

	Buried surface area (per strand)	Shape complementarity	Average solvation energy (ΔG°)/residue
M9I Dry interface	128.2 Å^2	0.880	-0.56 kcal/mol
M9I Hydrated interface	117.5 Å^2	0.314	-

Fig. S4: Crystal lattice formed by M9I

(**A**) Model of M9I crystal lattice, with one unit cell boxed and white mesh representing the $2F_o$-F_c density map contoured at 1σ. Red spheres represent ordered water molecules. Each unit cell contains 2 asymmetric units, with 1 peptide molecule in each asymmetric unit. (**B**) Model of M9I crystal lattice with surface representation, showing self-complementary interdigitation of C-

terminal side chains in a dry, tightly packed interface. In contrast, N-terminal side chains primarily form hydrogen-bonds (yellow dashed lines) with a network of ordered water molecules (red). (**C**) Calculated buried surface area and shape complementarity (Sc) for dry (C-terminal) and hydrated (N-terminal) interfaces, as well as the average solvation energy per residue of the dry interface.
